# Real-world effectiveness of lithium

**DOI:** 10.1192/j.eurpsy.2026.10394

**Published:** 2026-07-31

**Authors:** J. Tiihonen

**Affiliations:** 1 Clinical Neuroscience, Karolinska Institutet, Stockholm, Sweden; 2 Forensic Psychiatry, University of Eastern Finland, Kuopio, Finland

## Abstract

**Introduction:**

Lithium is considered the first-line treatment for bipolar disorder but its use has been declining worldwide. This is apparently attributable to its side effects and to the fact that lithium is inexpensive, meaning that pharmacological companies have no incentive for marketing.

**Objective:**

To give an overview on the real-world evidence on the effectiveness of lithium of bipolar disorder and unipolar depression.

**Methods:**

Literature review in PubMed.

**Results:**

All large real-world studies indicate that lithium is highly beneficial treatment compared with other options for bipolar disorder, concerning relapses, suicidality, working ability, and somatic well-being of the patients. It was observed also to be useful in the augmentation of treatment for unipolar depression.

**Conclusions:**

The results suggest the use of lithium as the mainstay of treatment in bipolar disorder.

**Disclosure of Interest:**

J. Tiihonen Grant / Research support from: EU/Forte, State Research Funding, Jane and Aatos Erkko Foundation; Research collaboration with Janssen and Lecture fees from Janssen, Lundbeck, Orion Pharma, Otsuka, and Teva, Consultant of: Healthcare Global Village, Janssen, Lundbeck, Orion Pharma, and Teva; Expert testimony for Janssen; Advisory Board member Teva.

